# Reducing Potentially Inappropriate Prescriptions for Older Patients Using Computerized Decision Support Tools: Systematic Review

**DOI:** 10.2196/15385

**Published:** 2019-11-14

**Authors:** Luís Monteiro, Tiago Maricoto, Isabel Solha, Inês Ribeiro-Vaz, Carlos Martins, Matilde Monteiro-Soares

**Affiliations:** 1 Esgueira+ Family Health Unit, Aveiro Healthcare Centre Aveiro Portugal; 2 Center for Health Technology and Services Research Faculty of Medicine University of Porto Porto Portugal; 3 Aveiro-Aradas Family Health Unit, Aveiro Healthcare Centre Aveiro Portugal; 4 Faculty of Health Sciences University of Beira Interior Covilhã Portugal; 5 Terras de Souza Family Health Unit Paredes Portugal; 6 Porto Pharmacovigilance Centre, Faculty of Medicine, University of Porto Porto Portugal; 7 Department of Community Medicine, Information and Decision in Health Faculty of Medicine University of Porto Porto Portugal

**Keywords:** deprescriptions, medical informatics applications, potentially inappropriate prescription, potentially inappropriate medication, computerized decision support

## Abstract

**Background:**

Older adults are more vulnerable to polypharmacy and prescriptions of potentially inappropriate medications. There are several ways to address polypharmacy to prevent its occurrence. We focused on computerized decision support tools.

**Objective:**

The available literature was reviewed to understand whether computerized decision support tools reduce potentially inappropriate prescriptions or potentially inappropriate medications in older adult patients and affect health outcomes.

**Methods:**

Our systematic review was conducted by searching the literature in the MEDLINE, CENTRAL, EMBASE, and Web of Science databases for interventional studies published through February 2018 to assess the impact of computerized decision support tools on potentially inappropriate medications and potentially inappropriate prescriptions in people aged 65 years and older.

**Results:**

A total of 3756 articles were identified, and 16 were included. More than half (n=10) of the studies were randomized controlled trials, one was a crossover study, and five were pre-post intervention studies. A total of 266,562 participants were included; of those, 233,144 participants were included and assessed in randomized controlled trials. Intervention designs had several different features. Computerized decision support tools consistently reduced the number of potentially inappropriate prescriptions started and mean number of potentially inappropriate prescriptions per patient. Computerized decision support tools also increased potentially inappropriate prescriptions discontinuation and drug appropriateness. However, in several studies, statistical significance was not achieved. A meta-analysis was not possible due to the significant heterogeneity among the systems used and the definitions of outcomes.

**Conclusions:**

Computerized decision support tools may reduce potentially inappropriate prescriptions and potentially inappropriate medications. More randomized controlled trials assessing the impact of computerized decision support tools that could be used both in primary and secondary health care are needed to evaluate the use of medication targets defined by the Beers or STOPP (Screening Tool of Older People’s Prescriptions) criteria, adverse drug reactions, quality of life measurements, patient satisfaction, and professional satisfaction with a reasonable follow-up, which could clarify the clinical usefulness of these tools.

**Trial Registration:**

International Prospective Register of Systematic Reviews (PROSPERO) CRD42017067021; https://www.crd.york.ac.uk/prospero/display_record.php?ID=CRD42017067021

## Introduction

The older adult population is increasing in developed countries [1], and people worldwide are living longer [2,3]. According to the World Health Organization, people aged 60 years and older in 2020 will outnumber children younger than 5 years. In 2050, the world’s population aged 60 years and older is expected to total 2 billion [2].

The aging of populations increases the pressure on health care systems, which should be aligned with the needs of older populations [4]. Older patients are more likely to have more than one chronic condition, known as multimorbidity [5,6]. The prevalence of multimorbidity is more than 90% in older patients [5]. Having more than one chronic condition requires the use of several medications. Thus, older adults are more vulnerable to polypharmacy [7], meaning the use of multiple drugs administered to the same patient [8,9], in addition to prescriptions of potentially inappropriate medications (PIMs) [10-12]. A PIM can be described as a medication use that has potentially more risks than benefits with a safer alternative available [10].

Potentially inappropriate prescription (PIP) is a broader concept than PIM, because it includes over-, under-, and misprescribing (eg, inappropriate dose or duration). It is defined as “the prescribing of medication that could introduce a significant risk of an adverse event, in particular when there is an equally or more effective alternative with lower risk available” [13].

Due to changes in pharmacokinetics and pharmacodynamics, older people are more prone to drug interactions and adverse drug reactions [14,15]. Adverse drug reactions are considered a public health problem in older patients and a cause of disability and mortality [15]. Deprescribing is defined as “the process of withdrawal of inappropriate medication, supervised by a health care professional, with the goal of managing polypharmacy and improving outcomes” [16].

There are several ways to address polypharmacy to prevent its occurrence [17-23]. This review focused on computerized decision support (CDS) tools. Bates et al [24] defined CDS systems as computer-based systems providing “passive and active referential information as well as reminders, alerts, and guidelines.” Payne [25] added that CDS tools can be defined as “computer applications designed to aid clinicians in making diagnostic and therapeutic decisions in patient care.” CDS tools may have a positive impact on health care, such as reducing physicians’ orders of unnecessary tests [26].

Previous studies reviewed such strategies, such as multidisciplinary team medication reviews, pharmacist medication reviews, computerized clinical decision support systems, and multifaceted approaches and reported substantial heterogeneity in the included studies, but did not focus on CDS [19,21]. One systematic review that did focus on CDS systems included studies published only through 2012, and new studies have been published since then [27]. This systematic review aims to clarify whether CDS tools can help in reducing PIPs or PIMs to improve clinical outcomes in older adults.

## Methods

### Eligibility Criteria

The systematic review was conducted according to a protocol previously published [28] and registered in PROSPERO (International Prospective Register of Systematic Reviews; CRD42017067021). We searched for interventional controlled studies (type of study) with participants aged 65 years or older (population) that assessed whether CDS tools (intervention) could diminish PIM (outcome). Moribund or terminal participants were excluded along with those requiring palliative care. No other restriction was applied.

### Search Methods

We searched MEDLINE, CENTRAL, EMBASE, and Web of Science for studies published through February 2018 without language restrictions. Specific queries were used according to each database’s requirements that were described in detail elsewhere [29]. Trial registries, different types of grey literature, and contact with specialists in the field were also performed. The reference lists of all included studies were searched to identify any potentially pertinent study that might not have been identified by previous methods. References were checked from previously published systematic reviews.

### Selection Process

Articles were selected by applying the criteria to the title and abstract of each study. Studies that were selected at this stage were then assessed in their entirety. Each stage was conducted by two researchers blindly and independently. Two reviewers (LM and TM) examined the titles and abstracts and did the full-text screening. When disagreement occurred, it was resolved through consensus.

### Data Collection Process

For all the included studies, characterization of data and results were exported into a datasheet by one of the authors (LM) and confirmed by the other (MS).

### Type of Data Collected

Studies were characterized according to setting, intervention, comparison definition, study duration, number of included participants overall and in each study group, the proportion of missing data, participants’ mean age, the proportion of male individuals, and deprescribing target. Outcomes retrieved from each study were categorized as PIP- or PIM-related and by overall number of prescriptions, adverse drug reactions, and potential drug-drug interactions.

### Analysis of Results and Assessment of the Risk of Bias

Possible bias in randomized controlled trials (RCTs) was independently identified using the Cochrane Collaboration Risk of Bias tool [29] by two researchers (TM and LM). This assessment was confirmed by other authors (IV and MS). Risk of bias was determined with regard to random sequence generation, allocation concealment, blinding of participants and personnel, blinding of outcome assessments, incomplete outcome data, selective outcome reporting, and other biases.

The included articles did not permit the performance of a meta-analysis because there were not a minimum of three studies using the same deprescribing target. Thus, only a narrative synthesis was performed. We have summarized the main features and results of all the included studies, discussed their limitations, and proposed future research avenues.

## Results

### Description of the Studies

**Figure 1:**
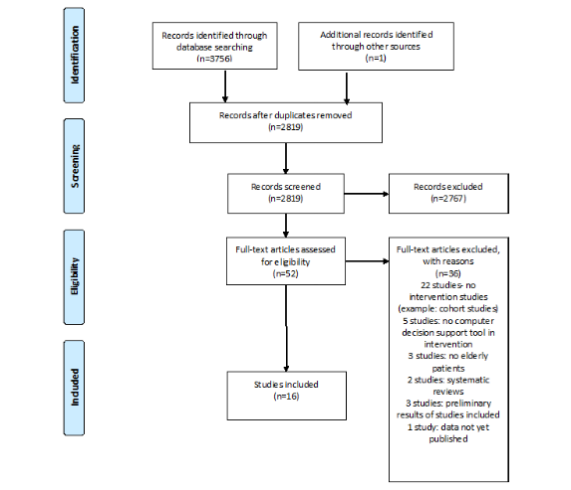
Flow diagram on search and article inclusion, according to the Preferred Reporting Items for Systematic Reviews and Meta-Analyses (PRISMA) Statement.

Using our search strategy, 3756 articles were identified through MEDLINE, Central, EMBASE, and Web of Science databases. One article was identified through contact with specialists. After duplicates were removed, 2819 articles remained. The titles and abstracts were screened, and 2767 studies were excluded. Of these, 52 articles were selected to assess eligibility and their full text was analyzed. Of these, 36 articles were excluded. Ultimately, we included 16 studies in our systematic review. No new article was found by searching in the included studies’ reference lists, trial registries, or grey literature. The article selection process and reasons for exclusion are described in Figure 1.

The characteristics of the included studies are described in Table 1. More than half (10/16) of the included studies were RCTs, one was a crossover study, and five were pre-post intervention studies. Most studies were conducted in North America (Canada and United States; n=11) [30-40]. The remaining were conducted in Europe (n=5) [41-45].

Six studies were conducted exclusively in secondary health care institutions [35,37,38,40,44,45]. In two studies, only emergency department participants were included [33,39]. In total, six studies were performed exclusively in primary health care institutions [30-32,41-43], one study took place in a health maintenance organization [34], and one study included participants from both secondary and primary health care institutions [36]. Six studies took place at teaching hospitals [36-38,40,44,45].

Most commonly, the standard of care was the only comparator (n=11). The interventional design was always based on a CDS tool, which was usually included in the electronic medical record with several different features. In some cases (n=6), complex interventions were performed that included training and engagement sessions and/or leaflet provision.

The RCTs had an inclusion period ranging from 3 to 30 months (see Table 2). The crossover study included four on-off periods with a 6-week duration [33]. The pre-post intervention studies frequently compared different time periods.

**Table 1:** Descriptions of the included studies in the systematic review (N=16).

Author, year; (study); country		Setting	Comparator	Intervention	Deprescribing target
**Randomized controlled trials**					
	Tamblyn et al [30], 2003; Canada	PHC^a^	Usual care^b^	Computerized decision support tool providing alert identified problem + presented possible consequences + provided alternative therapy	PIP^c^ (159 clinically relevant PIPs in the elderly defined by expert consensus)
	Price et al [31], 2017; Canada	PHC (8 GP^d^)	Usual care	Clinical decision support tool showing alert with specific STOPP^e^ guideline content in electronic medical record	PIPs (40 STOPP criteria)
	Avery et al [41], 2012; (PINCER); UK	PHC (72 GP)	Computer-generated simple feedback	PINCER; comparator + pharmacist-led information technology complex intervention	PIPs on NSAIDs^f^, beta blockers, ACE^g^ inhibitors, or loop diuretics
	Erler et al [42], 2012; Germany	PHC (46 GP)	Usual care	Interactive 1-hour workshop for physicians on detection and management of CKD^h^ + provision of desktop checklist of medications to be reduced or avoided + patient information leaflets + training in the use of software “DOSING”	Prescription exceeding recommended standard; daily dosage >30% or recommended; maximum daily dose in CKD patients
	Clyne et al [43], 2015; (OPTI-SCRIPT); Ireland	PHC (21 GP)	Usual care + simple, patient-level PIP postal feedback	Comparator + academic detailing with pharmacist + medicine review with Web-based pharmaceutical treatment algorithms + leaflets	PIPs using 28 criteria from the study
	Cossette et al [40], 2017; Canada	SHC^i^ (teaching hospital)	Usual care	KT^j^ strategy; distribution of educational materials + in-services by geriatricians + computerized alert systems pharmacist-physician	7 PIMs^k^ based Beers and STOPP geriatric criteria and drugs with anticholinergic properties or acting on the central nervous system
	Fried et al [32], 2017; (TRIM); USA	PHC (Veterans Affairs; medical center)	Usual care only and usual care with telephonic patient assessment	2 Web apps: (1) extracts information on medications and chronic conditions from the electronic health record, (2) interface for data chart review and telephonic patient assessment + a set of automated algorithms evaluating medication appropriateness + patient-specific medication management feedback report for the clinician	Medication appropriateness based on range of criteria, including feasibility in context of patient’s cognition and social support, potential overtreatment of DM^l^ or hypertension, “traditional” PIMs according to Beers and STOPP criteria, inappropriate renal dosing, and patient report of adverse medication effects
	O’Sullivan et al [44], 2016; Ireland	SHC (teaching hospital)	Usual medical and pharmaceutical care	Clinical decision support software supported structured pharmacist review of medication designed to optimize geriatric pharmaceutical care	Medicines associated with “nontrivial” adverse drug reactions (according to WHO)
	Terrel et al [33], 2009; USA	ED^m^ (teaching hospital)	Computerized; physician order entry without alerts	Computer-assisted decision support alert when PIM was being prescribed + rationale + recommended safer substitute therapies. If physician chose to continue, second menu displayed to query most important reason	9 high-use and high-impact PIMs^n^
	Raebel et al [34], 2007; USA	HMO^o^ (18 medical offices + 21 pharmacies)	Usual care	Medication alert generated from PIMS not allowing prescription label to be printed until the pharmacist actively determined whether prescription should be dispensed; pharmacists should communicate notifications to prescribing clinicians	Newly prescribed PIMs based on the Beers, Zhan and Kaiser Performance Care Management Institute lists of medications to be avoided in older people^p^
**Crossover studies**					
	Peterson et al [35], 2005; USA	SHC	Usual computerized order entry	Guided dosing of psychotropic medication integrated in Brigham Integrated Computer System	Benzodiazepines, opiates, and neuroleptics
**Pre-post intervention studies**					
	Ruhland et al [36], 2017; USA	SHC + PHC; (1 teaching hospital + 2 community hospital + 31 clinics)	Usual care	Clinical decision support system creating an alert + rational and; alternative medication through Epic (an integrated electronic medical record)	PIMs on glyburide
	Mattinson et al [37], 2010; USA	SHC (teaching hospital)	Usual care	Medication-specific warning system (advised alternative medication or dose reduction)	PIMs on medications not recommended for use in older patients (not recommended medications) and those for which only a reduced dose was advised (dose-reduction medications)
	Lester et al [38], 2015; USA	SHC (teaching hospital)	Computerized physician order entry without alerts	Computerized; physician order entry with pop-up alerts for selected PIPs containing links to articles relevant to the alert	PIPs on diphenhydramine, metoclopramide, and antipsychotics
	Ghibelli et al [45], 2013; (INTERcheck); Italy	SHC (teaching hospital)	Analysis without any interference	Computer-based application (INTERCheck) that collects, stores and automatically; provides drug information to reduce or prevent PIPs	PIMs from 2003 Beers Criteria; potential DDIs^q^; and Anticholinergic Cognitive Burden Scale
	Stevens et al [39], 2017; (EQUiPPED); USA	ED (10 Veterans Affairs; medical centers)	Usual care	EQUiPPED interventions: education + informatics-based clinical decision support + individual provider feedback	PIMs from 2012 Beers Criteria category 1 (to avoid in all older adults)

^a^PHC: primary health care.

^b^Each physician was given a computer, printer, health record software, and access to the internet.

^c^PIP: potentially inappropriate prescription.

^d^GP: general practice.

^e^STOPP: Screening Tool of Older People’s Prescriptions.

^f^NSAID: nonsteroidal anti-inflammatory drug.

^g^ACE: angiotensin-converting enzyme.

^h^CKD: chronic kidney disease.

^i^SHC: secondary health care.

^j^KT: knowledge translation.

^k^PIM: potentially inappropriate medication.

^l^DM: diabetes mellitus.

^m^ED: emergency department.

^n^High-use and high-impact PIMs: promethazine, diphenhydramine, diazepam, propoxyphene with acetaminophen, hydroxyzine, amitriptyline, cyclobenzaprine, clonidine, indomethacin.

^o^HMO: health maintenance organization.

^p^Examples of medications to be avoided in older people: amitriptyline, chlordiazepoxide, chlorpropamide, diazepam, doxepin, flurazepam, aspirin in combination with hydrocodone or oxycodone, ketorolac, oral meperidine, and piroxicam.

^q^DDI: drug-drug interaction.

**Table 2:** Characterization of the included studies in the systematic review, including study type, study duration, sample size, and participant demographics (N=16).

Study		Study duration (months); date range	Sample size, N	Participants, n			Outcome missing data, n (%)
					Age (years), mean (SD)	Gender (male), n (%)	
**Randomized controlled trials**							
	Tamblyn et al [30]	13; (01/1997-02/1998)	12,560	C^a^: 6276; I^b^: 6284	C: 75 (6); I: 75 (6)	C: 2248 (36); I: 2439 (39)	N/R^c^
	Price et al [31]	8; (02-10/2015)	81,905	C:37,615; I: 44,290	N/R; all >65 years	N/R	N/R
	Avery et al [41]	6 (and 12)	480,942	C: 37,659; I: 34,413	N/R	N/R	C: 22 (0.06); I: 28 (0.08) for outcome 3
	Erler et al [42]	6	404	C: 206; I: 198	C: 80 (9); I: 81 (6)	C: 63 (31); I: 81 (41)	C: 9 (4); I: 0 (0)
	Clyne et al [43]	6; (10/2012-09/2013)	196	C: 97; I: 99	C: 76 (5); I: 77 (5)	C: 50 (52); I: 55 (56)	C: 3 (3); I: 3 (3)
	Cossette et al [40]	10 weeks; (09/2015-12/2015)	321	C: 133; I: 139	C: 81 (7); I: 82 (8)	C: 53 (41); I:48 (38)	C: 5 (4); I: 13 (9)
	Fried et al [32]	3; (10/2014-01/2016)	156	C1: 36; C2: 39; I: 81	<70 years C: 25 (39); I: 27 (42)	C: 63 (99); I: 63 (99)	C1: 4 (11); C2:7 (18); I: 17 (21)
	O’Sullivan et al [44]	13; (06/2011-07/2012)	737	C: 361; I: 376	C: 78b; (IQR 72-84); I: 77; (IQR 71-83)	C: 190 (51); I: 180 (50)	C: 17 (5); I: 17 (5)
	Terrel et al [33]	30; (12/01/2005-07/07/2007)	5162	C: 2515; I: 2647	C: 74 (7); I: 74 (7)	C: 880 (35); I: 929 (35)	N/R
	Raebel et al [34]	12; (18/05/2005-17/05/2006)	59,680	C: 29,840; I: 29,840	C: 74; (5-95 percentile 66-88); I: (5-95 percentile 66-88)	C: 12,843 (43); I: 12704 (43)	N/R
**Crossover studies**							
	Peterson et al [35]	4 × 6 week on-off periods; (08/10/2001-16/05/2002)	3718	C: 1925; I: 1793	C: 75 (7); I: 75 (7)	C: 905 (47); I: 843 (47)	N/R
**Pre-post intervention studies**							
	Ruhland et al [36]	3 + 3; (B^d^: 01/12/2014-28/02/2015); A^e^: 01/03/2015-31/05/2015)	N/R	101 patients with activated alert	75	N/R	N/A^f^
	Mattison et al [37]	6 + 41.5; (B: 1/06-29/11/2014; A: 17/03/2015-30/08/2008)	N/R	N/R	N/R; all >65 years	N/R	N/R
	Lester et al [38]	12 + 24; (B: Q2 2010; A: Q2s 2011-2013)	29,465	B: 6604; A: 22,861	<75 years; B: 5279 (80); A: 15,633 (68)	N/R	N/R
	Ghibelli et al [45]	2 + 2; (B: 04 to 05/2012; A: 06 to 07/2012)	134	B: 74; A: 60	B: 81; A: 81	B: 27 (36); A: 25 (42)	B: 0 (0); A: 0 (0)
	Stevens et al [39]	>6 + >12	N/R	N/R	N/R; all >65 years	N/R	N/R

^a^C: comparator group.

^b^I: intervention group.

^c^N/R: not reported.

^d^B: before.

^e^A: after.

^f^N/A: not applicable.

A total of 233,144 participants were included and assessed in RCTs (mean sample size: 21,199; range 196-72,072 participants). The crossover study included 3718 individuals. The pre-post intervention studies included more than 29,700 participants. However, some studies did not report a raw number of participants included in each study period. There was no information regarding whether missing data influenced the outcome assessment in eight studies (50%).

According to our inclusion criteria, all individuals were older than 65 years of age. The mean age in the selected studies was approximately 75 years. Females were often more prevalent, especially in larger studies.

The deprescribing target varied among the studies, and several papers used more than one criterion [30,32-34,40,45]. PIM was defined in some papers using internationally recognized criteria, such as the Beers Criteria (n=5) [32,34,39,40,45], the Screening Tool of Older People’s Prescriptions (STOPP) criteria (n=3) [31,32,40], and the Anticholinergic Cognitive Burden Scale (n=1) [45]. In other studies (n=4), some group medications were specifically the target, such as benzodiazepines, opiates, and neuroleptics [35]; glyburide [36]; nonsteroidal anti-inflammatory drugs (NSAIDs), beta blockers, angiotensin-converting enzyme (ACE) inhibitors, or loop diuretics [41]; and diphenhydramine, metoclopramide, and antipsychotics [38].

### Results of the Studies

The main results of the included studies are described in Tables 3 and 4. Several definitions and units were used to measure the impact of CDS tools on changes in PIP and PIM drugs (overall or concerning specific drugs). Studies assessed the following PIP- or PIM-related outcomes: number of PIMs started per 1000 visits [30], number of PIMs discontinued per 1000 visits [30], proportion of discontinued PIMs [30], percentage of PIMs [43], mean number of PIMs, risk of receiving a prescription for a drug exceeding the recommended maximum dose [42], risk of receiving a prescription for a drug exceeding the recommended standard doses [42], proportion of reconciliation errors corrected [32], proportion of recommendations implemented [32,33], proportion of patients with at least one PIM, and/or proportion of all prescribed medications that were PIM [33].

**Table 3:** Results of the included studies including changes in potentially inappropriate prescriptions or medications (N=16).

Study		PIP^a^- or PIM^b^-related outcomes	
		Changes in PIP or PIM drugs	Changes in specific PIP or PIM drugs
		
**Randomized controlled trials**			
	Tamblyn et al [30]	Number of PIP started per 1000 visits C^c^: 52.2 vs I^d^: 43.8, RR^e^ 0.82 (CI^f^ 95% 0.69 −0.98); PIP discontinuation C: 44.5% vs I: 47.5%, RR: 1.14 (95% CI 0.98-1.33); number of PIP discontinued per 1000 visits C: 67.4 vs I: 71.4, RR 1.06 (95% CI 0.89-1.26)	Number of PIP started per 1000 visits: drug-disease contraindication C: 18.4 vs I: 16.6, RR 0.89 (CI 95% 0.72-1.10); drug-age contraindication C: 13.7 vs I: 10.7, RR 0.77 (CI 95% 0.59-1.00); excessive duration therapy C: 17.1 vs I: 13.3, RR 0.78 (CI 95% 0.61-0.99); therapeutic duplication C: 6.8 vs I: 6.1, RR 0.87 (CI 95% 0.69-1.11); number of PIP discontinued per 1000 visits: drug-disease contraindication C: 57.9 vs I: 62.6, RR 1.08 (CI 95% 0.85-1.36); drug-age contraindication C: 42.9 vs I: 40.7, RR 0.94 (CI 95% 0.79-1.13); excessive duration therapy C: 32.6 vs I: 32.3, RR 1.00 (CI 95% 0.77-1.29); therapeutic duplication C: 334.0 vs I: 317.1, RR 0.94 (CI 95% 0.59-1.51)
	Price et al [31]	Change in PIP C: 0.1% vs I: 0.1%, *P*=.80	
	Avery et al [41]	—^g^	At 6 months: history of peptic ulcer prescribed an NSAID^h^ without a PPI/history of peptic ulcer without PPI^i^ AOR^j^ 0.58 (95% CI 0.38-0.89); asthma prescribed a β blocker/asthma AOR 0.73 (95% CI 0.58-0.91); aged ≥75 years long-term ACE^k^ inhibitors or loop diuretics without urea and electrolyte monitoring in the previous 15 months aged ≥75 years receiving long-term ACE inhibitors or diuretics AOR 0.51 (95% CI 0.34-0.78); secondary outcomes AOR varied from 0.39-0.96; at 12 months: history of peptic ulcer prescribed an NSAID without a PPI/history of peptic ulcer without PPI AOR 0.91 (95% CI 0.59-1.39); asthma prescribed a β blocker/asthma AOR 0.78 (95% CI 0.63-0.97); aged ≥75 years receiving long-term ACE inhibitors or loop diuretics without urea and electrolyte monitoring in the previous 15 months aged ≥75 years receiving long-term ACE inhibitors or diuretics AOR 0.63 (95% CI 0.41-0.95); secondary outcomes AOR varied from 0.50-0.98
	Erler et al [42]	CKD^l^ patients with ≥1 prescription exceeding recommended maximum dose AOR 0.46 (95% CI 0.26-0.82); CKD patients with ≥1 prescription exceeding recommended standard dose by >30% AOR 0.66 (95% CI 0.36-1.21)	NS differences in the numbers of patients with potentially dangerous or contraindicated medications
	Clyne et al [43]	Percentage of PIP I: 52% vs C: 77%, *P*=.02, AOR 0.32 (95% CI 0.15-0.70); mean number of PIP C: 1.18 vs I: 0.70, *P*=.02	Odds of PIP AOR 0.30 (95% CI 0.14-0.68); NS differences for duplicate or long-term benzodiazepines
	Cossette et al [40]	Drug cessation or dosage decrease: at 48h C: 15.9% vs 45.8%, AD^m^ 30.0% (95% CI 13.8-46.1); at discharge C: 27.3% vs I: 48.1%, AD 20.8% (95% CI 4.6-37.0); drug cessation: at 48h C: 15.1% vs 51.9%, AD 36.8% (95% CI 15.6-57.9); at discharge C: 34.4% vs I: 45.2%, AD 10.7% (95% CI −10.5 to 31.9); dosage decrease: at 48h C: 17.2% vs 38.1%, AD 20.9% (95% CI 4.1-45.8); at discharge C: 15.8% vs I: 52.4%, AD 36.6% (95% CI 12.3-60.9)	—
	Fried et al [32]	Proportion of medication reconciliation errors corrected C: 14.3% vs I: 48.4%, *P*<.001; proportion of ≥1 TRIM recommendations implemented C: 21.9% vs I: 29.7%, *P*=.42	—
	O’Sullivan et al [44]	Patients with ≥1 PIP C: 84.6% vs I: 82%	—
	Terrel et al [33]	Proportion of visits with a PIP C: 3.9% vs I: 2.6, *P*=.02, OR^n^ 0.55 (95% CI 0.34-0.89), ARR^o^ 1.3% (95% CI 0.4-2.3); proportion of all prescribed medications that were PIP C: 5.4% vs I: 3.4, *P*=.006, OR 0.59 (CI 95% 0.41-0.85), ARR 2.0% (95% CI 0.7-3.3)	—
	Raebel et al [34]	Newly dispensed ≥1 PIP rate per 100 patients C: 2.20 vs I:1.85, *P*=.002, RRR^p^ 16%; newly dispensed ≥1 PIP only for indications included in intervention rate per 100 patients C:1.50 vs I: 1.10, *P*<.001	Newly dispensed ≥1 PIP rate per 100 patients: amitriptyline C: 0.61 vs I: 0.38, *P*<.001; chlordiazepoxide C: 0.05 vs I: 0.04, *P*=.55; diazepam C: 1.38 vs I: 1.28, *P*=.32; doxepin C: 0.14 vs I: 0.11, *P*=.24; flurazepam C: 0.01 vs I: 0.01, *P*=.69; ketorolac C: 0.00 vs I: 0.01, *P*=.50; meperidine (oral) C: 0.01 vs I: 0.01, *P*=N/A^q^; oxycodone/aspirin C: 0.00 vs I: 0.00, *P*=N/A; newly dispensed ≥1 PIP only for indications included in intervention, rate per 100 patients: amitriptyline C: 0.59 vs I: 0.37, *P*<.001; chlordiazepoxide C: 0.05 vs I: 0.04, *P*=.55; diazepam C: 0.71 vs I: 0.56, *P*=.002; doxepin C: 0.13 vs I: 0.09, *P*=.17; flurazepam C: 0.01 vs I: 0.01, *P*=.69; ketorolac C: 0.00 vs I: 0.01, *P*=.50; meperidine (oral) C: 0.01 vs I: 0.01, *P*=N/A; oxycodone/aspirin C: 0.00 vs I: 0.00, *P*=N/A; dispensings of chlorpropamide, hydrocodone/aspirin, or piroxicam C: 0 vs I: 0
**Crossover studies**			
	Peterson et al [35]	Prescription recommended daily dose C: 19% vs I: 29%, *P*<.001; prescription orders with 10-fold dosing C: 5.0% vs I: 2.8%, *P*<.001; prescriptions in agreement with recommendation C: 18.6% vs I: 29.3%, *P*<.001; prescription of nonrecommended drugs C: 10.8% vs I: 7.6%, *P*<.001	Prescription orders with 10-fold dosing: benzodiazepines C: 3.5% vs I: 2.0%, *P*=.01; opiates C: 5.5% vs I: 2.8%, *P*<.001; neuroleptics C: 10.0% vs I: 7.5%, *P*=.35; prescriptions in agreement with recommendation: benzodiazepines C: 20.8% vs I: 28.2%, *P*<.001; opiates C: 16.6% vs I: 29%, *P*<.001; neuroleptics C: 22.5% vs I: 38%, *P*<.001
**Pre-post intervention studies**			
	Ruhland et al [36]	—	Glyburide orders from total oral antidiabetic orders B^r^: 3.3% vs A^s^: 1.6%, *P*<.001; 17.8% patients transitioned off glyburide
	Mattison et al [37]	Number of orders per total number of patients per day: not recommended medication B: 0.070 vs A: 0.054, *P*<.001; dose reduction medications B: 0.037 vs A: 0.037, *P*=.71; unflagged medications B: 0.033 vs A: 0.030, *P*=.03; number of orders per number of new patients per day: not recommended medication B: .333 vs A: 0.263, *P*<.001; dose reduction medications B: 0.182 vs A: 0.186, *P*=.51; unflagged medications B: 0.158 vs A: 0.148, *P*=.08	—
	Lester et al [38]	—	>65 years prescription rates of: diphenhydramine B: 26.9% vs A: 20%, *P*<.001; metoclopramide B: 16.7% vs A: 12.5%, *P*<.001; antipsychotics B: 8.8% vs A: 9.2%, *P*=.80; ≥65 years: no significant changes for diphenhydramine, metoclopramide, or antipsychotics
	Ghibelli et al [45]	Proportion of patients exposed to PIM at discharge B: 37.8% vs A: 11.6%; mean number of PIM per patient at discharge B: 0.4 vs A: 0.1	Proportion of patients exposed to PIM at discharge: high-dose short-acting benzodiazepines B: 21.6% vs A: 6.7%; ticlopidine B: 5.4% vs A: 0.0%; digoxin B: 5. 4% vs A: 1.7%; doxazosin B: 1.3% vs A: 1.7%; clonidine B: 1.3% vs A: 0.0%
	Stevens et al [39]	Average percentage of PIMs per month: site 1 B: 11.9 vs A: 5.1, *P*<.001; site 2 B: 8.2 vs A: 4.5, *P*<.001; site 3 B: 8.9 vs A: 6.1, *P*=.007; site 4 B: 7.4 vs A: 5.7, *P*=.04	—

^a^PIP: potentially inappropriate prescription.

^b^PIM: potentially inappropriate medication.

^c^C: comparator group.

^d^I: intervention group.

^e^RR: relative rate.

^f^CI: confidence interval.

^g^No data.

^h^NSAID: nonsteroidal anti-inflammatory drug.

^i^PPI: proton-pump inhibitor.

^j^AOR: adjusted odds ratio.

^k^ACE: angiotensin-converting enzyme.

^l^CKD: chronic kidney disease.

^m^AD: absolute difference.

^n^OR: odds ratio.

^o^ARR: absolute risk reduction.

^p^RRR: relative risk reduction.

^q^N/A: not applicable.

^r^B: before.

^s^A: after.

**Table 4:** Results of the included studies including number of prescriptions, adverse drug reactions, and potential drug-drug interactions (N=16).

Study		Overall number of prescriptions	Adverse drug reaction	PDDI^a^	Others
**Randomized controlled trials**					
	Tamblyn et al [30]	— ^b^	—	Number of PDDI started per 1000 visits C^c^: 1.5 vs I: 1.6, RR^d^ 1.12 (CI^e^ 95% 0.68-1.87); number of PPDI discontinued per 1000 visits C: 68.6 vs I^f^: 51.5 per 1000 visits, RR 1.33 (CI 95% 0.90-1.95)	Physicians with more computer problems downloaded information less often (*r*=−.31)
	Price et al [31]	—	—	—	Description of 12 data quality probes; alert awareness: all participants in I were aware of STOPP^g^ alerts, but not consistently; workflow and display: location on screen and workflow identified as barriers; study disruptiveness: considered as minimal
	Avery et al [41]	—	—	—	Mean ICER^h^ of intervention: at 6 months ₤65.6 (2.5-97.5 percentile 58.2-73.0); at 12 months ₤66.5 (2.5-97.5 percentile 66.8-81.5)
	Erler et al [42]	—	—	—	—
	Clyne et al [43]	—	—	—	Beliefs about Medicine Questionnaire AOR^i^ 0.16 (CI 95% −1.85 to 1.07); 12-item Well-Being Questionnaire AOR −0.41 (95% CI −0.80 to 1.07)
	Cossette et al [40]	—	—	—	LOS^j^ (median, IQR^k^) C: 9.5 (5-21) vs I: 10 (6-19), *P*=.9; in-hospital death C:11 (8.6%) vs I: 6 (4.8%), *P*=.3; 30-day post discharge ER visits C: 27 (21.1%) vs I: 27 (21.4%); 30-day postdischarge readmissions C: 28 (21.9%) vs I: 20 (15.9%), *P*=.3
	Fried et al [32]	Mean number of medications per patient C: 13.8 vs I: 13.3, *P*=.65	—	—	Mean patient active participation C: 2.7 vs I: 5.5, *P*=.001; percentage of patients assessment of care for chronic conditions score >10 C: 15.6% vs I: 29.7%, *P*=.06, OR^l^ 2.73 (CI 95% 0.82-9.08); patient medication-related; communication C: 3.6 vs I: 7.5, *P*<.001; mean clinician facilitative communication C: 0.67 vs I: 1.53, *P*=.02; mean clinician medication-related communication C: 4.6 vs I:7.3, *P*=.002; percentage >1 recommendations C: 32.8% vs I: 63.6%, *P*<.001; OR 3.33 (95% CI 1.37-8.04)
	O’Sullivan et al [44]	Total number of medications C: 3747 vs I: 4192, *P*<.001; median (IQR) number of medications per patient C: 9 (7-12) vs 12 (8-15), *P*<.001; number (%) of people with polypharmacy (≥5 medications); C: 346 (92.0) vs I: 346 (95.8), *P*=.44	Patients with ≥1 ADR^m^ C: 20.7% vs I: 13.9%, *P*= 0.02, ARR^n^ 6.8% (95% CI 1.5-12.3); RRR^o^ 33.3% (95% CI; 7.7-51.7); NNT^p^ 15 (95% CI 8-68)	—	CDS^q^ alerts 1000 in 296/361 patients; intervention group attended 54.8% of recommendations; median (IQR) LOS days C: 9 (5-16) vs I: 8 (5-13.5), *P*=.44; hospital mortality C: 4.5% vs I: 4.7%, *P*>.05; interrater reliability for application of WHO-UMC^r^ ADR causality criteria k= 0.81; Hallas ADR preventability criteria k= 0.87; application of Hartwig ADR; severity criteria k=0.56
	Terrel et al [33]	—	—	—	CDS alerts 114 during 107 visits; 43% of recommendations accepted
	Raebel et al [34]	—	—	—	—
**Crossover studies**			—	—	—
	Peterson et al [35]	Median (IQR) orders per admission C: 2 (1-3) vs I: 4 2 (1-3), *P*=.43	—	—	Number of altered mental status per 100 patient-days C: 21.9 vs I: 20.9, *P*=.17; median (IQR) LOS days C: 4 (2-6) vs I: 4 (2-6), *P*=.43; in-hospital fall rate C: 0.64 vs I: 0.28; falls per 100 patient-days, *P*<.001, AOR 0.50 (95% CI 0.30-0.82); fall injuries per 100 patient-days rate C: 0.17 vs I: 0.06, *P*=.09
**Pre-post intervention studies**			—	—	—
	Ruhland et al [36]	—	—	—	CDS tool alerted 101 times for 75 providers during encounters for 76 patients over 90 days; physicians were more likely to transition patients off glyburide vs other health care providers (46.2% vs 8.0%, *P*<.001)
	Mattison et al [37]	—	—	—	—
	Lester et al [38]	—	—	—	—
	Ghibelli et al [45]	—	—	Proportion of patients exposed to PDDI at discharge B^s^: 87.8% vs A^t^: 88.3%; mean number of PDDI per patient at discharge B: 4.5 vs A: 3.7	Median anticholinergic burden at discharge B: 1.5 vs A: 1.1
	Stevens et al [39]	—	—	—	—

^a^PDDI: potential drug-drug interactions.

^b^No data.

^c^C: comparator group.

^d^RR: relative rate.

^e^CI: confidence interval.

^f^I: intervention group.

^g^STOPP: Screening Tool of Older People’s Prescriptions.

^h^ICER: incremental cost-effectiveness ratio.

^i^AOR: adjusted odds ratio.

^j^LOS: length of stay.

^k^IQR: interquartile range.

^l^OR: odds ratio.

^m^ADR: adverse drug reaction.

^n^ARR: absolute risk reduction.

^o^RRR: relative risk reduction.

^p^NNT: number needed to treat.

^q^CDS: computerized decision support.

^r^UMC: Uppsala Monitoring Centre.

^s^B: before.

^t^A: after.

### Effects of Interventions

The CDS tools consistently reduced the number of PIPs started and the mean number of PIPs per patient, while also increasing PIM discontinuation and drug appropriateness. However, in several cases statistical significance was not achieved for some of the assessed measures, such as for PIM discontinuation in the Tamblyn et al article [30], for change in PIMs in the Price et al study [31], and other studies described in Table 3.

### Number of Prescriptions

With regard to the impact on the number of prescriptions, the RCT described by Fried et al [32] reported no significant reduction in the mean number of prescriptions in the group exposed to two Web apps. One study obtained information on medications and chronic conditions from an electronic health record, and the second study used an interface for data chart review, a telephone-based patient assessment, a set of automated algorithms evaluating medication appropriateness, and a patient-specific medication management feedback report for the clinician. In a crossover study [35], there were no significant differences in the median number of medications prescribed per patient during the periods in which guided dosing of psychotropic medication was integrated into the Brigham Integrated Computer System.

In contrast, the RCT described by O’Sullivan et al [44] demonstrated that those in the intervention group (using CDS software structuring pharmacist review of medications designed to optimize geriatric pharmaceutical care) prescribed significantly fewer drugs (both total and median number of drugs). However, no impact was observed for the proportion of people with polypharmacy prescribed more than five drugs at once. This RCT was the only one addressing adverse drug reactions and it concluded that using this software significantly reduced the risk of adverse drug reactions. Furthermore, only 15 patients’ medications needed to be reviewed to prevent one adverse drug reaction.

### Number of Potential Drug-Drug Interaction

Only two studies assessed whether CDS tools could decrease the number of potential drug-drug interactions [30,44]. One CDS used in an RCT was found to decrease the initiation of PIP, but it did not have a similar impact on deprescription [30].

One pre-post intervention study observed that the proportion of patients exposed to potential drug-drug interactions increased after implementing a computer-based app that collects, stores, and automatically provides drug information to reduce or prevent PIPs [45]. However, the mean number of potential drug-drug interactions per patient at discharge was reduced. Statistical significance was not reported.

### Other Measures

Other miscellaneous measures were reported in the studies examined, which should be highlighted. One RCT concluded that having computer problems was directly linked with PIP or PIM information download, and these computer problems could have an impact on the success of CDS tools [30]. Only one study described data quality probes; it found that professionals included in the intervention group were aware of STOPP alerts, although not in a consistent manner. Furthermore, the layout and impact on the workflow of the CDS tool were potential barriers to successful adherence [31].

### Adherence to Computerized Decision Support Tools

Several RCTs reported the frequency of adherence to CDS recommendations by a health professional, with values ranging from 33% to 55% [32,33,44]. No significant reduction in the length of stay or intrahospital mortality was found in the RCT described by O’Sullivan et al [44]; in the Cosstte et al study [40], the differences between the intervention and control groups were not statistically different. Similarly, a crossover study found no difference in the length of stay between periods when the CDS tool was either active or inactive [35]. Likewise, no difference was observed with respect to patients’ altered mental status or fall injuries. However, there was a significant decrease in the in-hospital rate.

The TRIM RCT concluded that the use of CDS tools significantly improved patients’ active participation and facilitated communication between the clinician and the patient [32]. Another RCT found no significant impact on the Beliefs about Medicine Questionnaire or the 12-item Well-Being Questionnaire when general practitioners had access to information from a pharmacist and a medical review with Web-based pharmaceutical treatment algorithms and leaflets in addition to the usual care and simple, patient-level PIP postal feedback [43].

### Cost-Effectiveness of Computerized Decision Support Tools

The cost-effectiveness of CDS tools was addressed in one RCT. The authors reported that there was a 95% probability that adding a pharmacist-led information technology complex intervention, in addition to computer-generated simple feedback, could be cost-effective, resulting in a willingness to pay ₤75 per error avoided at 6 months [41].

### Risk of Bias in the Studies Examined

The RCTs received a total score according to the Cochrane Collaboration Risk of Bias tool that ranged from 1 [30,31] to 5 [41,43]. The procedure to guarantee allocation concealment was unclear in eight of ten RCTs. Complete blinding of participants and personnel was not possible due to the nature of the intervention. Blinding for the outcome assessment was not conducted in five studies [31,34,40,41,44], and was unclear if it was successful in another two [30,42]. Both of these biases may have resulted in an overestimate of the CDS tools’ impact on PIP or PIM reduction (see Table 5).

**Table 5:** Risk of bias assessment (according to Cochrane Collaboration Risk of Bias tool) for the randomized controlled trials (n=10).

Study	Risk of bias items							Total score (max=7)
	Random sequence generation	Allocation concealment	Blinding of participants and personnel	Blinding of outcome assessment	Incomplete outcome data	Selective reporting	Other bias	
Tamblyn et al [30]	?^a^	?	–^b^	?	?	+^c^	–	1
Price et al [31]	+	?	–	–	?	?	–	1
Avery et al [41]	+	+	–	–	+	+	+	5
Erler et al [42]	+	?	–	?	+	+	–	3
Clyne et al [43]	+	?	–	+	+	+	+	5
Cossette et al [40]	+	?	–	–	–	-	+	2
Fried et al [32]	–	–	–	+	+	+	?	3
O’Sullivan et al [44]	?	?	–	–	+	+	–	2
Terrel et al [33]	+	?	–	+	?	+	–	3
Raebel et al [34]	+	?	–	–	?	+	+	3

^a^?: unclear risk of bias.

^b^–: high risk of bias.

^c^+: Low risk of bias.

Several studies did not report whether outcome data were available for all the participants included (n=4) [30,31,33,34]. Other biases were also found in five of the RCTs; namely, selection bias, performance bias, contamination, and underpowered sample sizes.

Regarding the pre-post intervention studies [36-39,45], they were considered high risk following the Cochrane Effective Practice and Organisation of Care [46]. For example, it is expected that pre-post intervention studies are more prone to the Hawthorne effect [47]. The Hawthorne effect happens when people (in this case, prescribers and patients) know they are being watched, which may lead to changes in behavior [47]. We consider that it is possible that being aware of one’s study participation could have resulted in prescribers taking more care when prescribing medications.

Limited generalizability was also pointed out by several authors as a major limitation due to the context—single-center design—and the use of CDS tools that were created specifically for the study, which may not be available in other institutions.

## Discussion

### Principal Results

Despite the fact that withdrawal of PIPs is considered to be evidence-based [48], it is not an easy task [49]. CDS tools may play a role in supporting deprescription. From the 16 studies examined in this review, 10 were RCTs. Although RCTs represent stronger evidence, they lacked important data pertaining to clinical outcomes and presented a significant risk of bias (the total score of the studies using the Cochrane Collaboration Risk of Bias tool ranged from 1 to 5 with a mean value of 3). The most frequent biases included no blinding of health professionals and an unclear risk of breaking allocation concealment. If prescribers are not blinded, this can easily affect the deprescribing process. Health professionals may have been more susceptible to accepting the CDS tool recommendations. Alternatively, patients may have been more likely to agree with the withdrawal process. If a break in allocation concealment occurred, it is expected that investigators may have potentially included older adults that they considered best suited for the intervention group. Both types of bias may have led to an overestimation of the benefit of CDS tools.

We have also included five pre-post intervention studies. The nonrandomized nature of these studies is the major limitation of this analysis. The impact of CDS tools may be confounded by other changes that may have occurred in the institutions during the study periods.

We observed that almost two-thirds of the included studies were performed in the United States, and one-third were performed in European countries. This reflects the importance that has been given to this topic only in developed countries where electronic health record systems are widely available.

### Overall Applicability and Quality of the Evidence

Seven studies were conducted in teaching hospitals and clinics [33,36-38,40,44,45], which may indicate potential bias. Teaching units are more prone to accept interventions in patient care, such as changes in a prescription through the use of CDS tools. We can assume that these professionals may be more likely to change a patient’s prescription and, therefore, to address PIPs. This tendency may result in an overestimate of the impact of the intervention, and we can only speculate as to what would be the impact in a nonteaching unit.

There is a balance between the number of studies conducted in primary care versus secondary care institutions, and only one was conducted in both. The impact of CDS on PIP or PIM reduction was similar between settings despite differences in the health professional and population characteristics. This suggests that the CDS tool might be successful in the context of a larger patient population.

The generalization of our results may be limited for several reasons. First, most studies used standard care as a comparator without providing additional details. In such a complex context, the management of older patients in institutions with several levels of care may mean that standard care could differ greatly between studies.

Second, the intervention varied greatly as a result of using different electronic systems, contents, and layouts. The intervention frequently included several features beyond the creation and application of a CDS tool itself.

Third, the main outcome definition was also diverse. Several studies used STOPP [31,32,40] and Beers Criteria [32,34,39,40,45] to define which medications were targeted. Both criteria are widely used worldwide, and although they do not provide a list of prohibited medications, they are an important tool for physicians due to their evidence-based rationale and constant updating. Nevertheless, the authors chose different groups of criteria for their outcome measures.

Fourth, the studies selected different participants and had widely variable sample sizes. Only two studies addressed potential drug-drug interactions [30,45] and one addressed adverse drug reactions [44]. Due to the increase of polypharmacy in older adults, the risk is higher for experiencing drug-drug interactions and adverse drug reactions. For the former, no significant impact was found, whereas for the latter, using a CDS tool significantly decreased the number of adverse drug reactions.

This tool, which included a clinical decision support software and a structured pharmacist review of medication [44], seems to be promising for aiding medication reconciliation activities. Most of the reconciliation issues highlighted by this CDS tool were accepted by the health care professionals involved. In particular, the Erler et al study [42] should, in our opinion, have assessed these two topics because they studied a population with renal impairment, which is particularly susceptible to adverse drug reactions and drug interactions. Similarly, only two studies assessed the impact of CDS tools on length of stay [35,40], and two assessed intrahospital mortality [40,44]. No differences were found between those using a CDS tool and those not using a CDS tool. Cost-effectiveness was also assessed by one study, which reported a 95% probability of a CDS tool being cost-effective due to a willingness to pay ₤75 to prevent an adverse drug reaction in a 6-month period [41]. The study’s results may have been underestimated due to low adherence to CDS recommendations. Three RCTs that evaluated adherence reported values fluctuating from 33% to 55% [32,33,44]. Finally, we consider the possibility that the Avery et al trial [41] could have explored the issue of prescription NSAIDs to patients with a history of asthma as a secondary outcome because the authors had information on both conditions (prescriptions of NSAIDs and a history of asthma). This analysis could yield interesting information about the patterns of prescribing NSAIDs to these patients.

### Strengths and Limitations

This review presents some limitations. We have chosen to include both RCTs (n=10) and pre-post studies (n=6). We acknowledge that the latter provide a lower level of evidence. Nevertheless, they have assessed some outcomes for which no additional evidence exists. In addition, we have focused our search on articles having PIP modification outcomes, thus some studies assessing changes in PIM may have been missed.

Our search terms were more limited to PIP; therefore, this paper may have missed some studies regarding PIM. Nevertheless, no new articles were found when searching in the references from the included studies and in the grey literature

Major strengths of our study include the fact that we have followed the Cochrane Collaboration Handbook [50], which makes our study less susceptible to major biases and errors. Furthermore, no new references were found from searches in the grey literature, pertinent scientific meeting books of abstracts, and the included studies’ list of references, which suggests that our search strategy was exhaustive and all pertinent articles had been included.

However, the quality of the results of a systematic review is dependent on the available data. For all that was previously described, we believed that conducting a meta-analysis was not possible. Thus, only a narrative synthesis has been provided.

### Comparison With Prior Work

To our knowledge, there are three previously published systematic reviews assessing the impact of CDS tools on PIP or PIM [51-27]. Due to an increase in the search period, the use of broader search criteria, and our overall methodology, we were able to include five additional RCTs [31,32,40,43,44]. These studies added evidence with new outcomes, such as well-being and patients’ beliefs [43], reduction of adverse drug reactions [44], and users’ perspectives [31].

The highlight of the findings in the more recent RCTs were as follows. In the study by Price et al [31], alerts with specific STOPP guideline content in electronic medical records positively changed PIPs (comparator: 0.1% versus intervention: 0.1%, *P*=.80), but not significantly. In the study by Clyne et al [43], the intervention consisted of Web-based pharmaceutical treatment algorithms that led to a lower percentage of PIPs (intervention: 52% versus comparator: 77%, *P*=.02). In the trial by Cossette et al [40], a computerized alert system-based pharmacist-physician intervention was able to significantly increase drug cessation or decrease dosage at discharge (comparator: 27.3% versus intervention: 48.1%; absolute difference 20.8%, 95% CI 4.6-37.0). In the TRIM trial [32], the proportion of medication reconciliation errors was significantly diminished (comparator: 14.3% versus intervention: 48.4%, *P*<.001). In the article by O’Sullivan et al [44], clinical decision support software reduced adverse drug reactions among older patients (control patients: 20.7% versus intervention patients: 13.9%, *P*=.02). In sum, articles published since 2012 substantiated the value of CDS to improve PIP- or PIM-related outcomes.

### Conclusions

The use of CDS tools had a positive impact on PIP independently of the outcome definition in the majority of the studies included in our analysis. However, statistical significance was not always achieved. Several possible sources of bias and experimental limitations were found in the included studies, and evidence is lacking regarding the impact of CDS tools in potential drug-drug interactions, adverse drug reactions, length of stay, mortality, and cost-effectiveness.

This research suggests that RCTs assessing the impact of CDS tools could be conducted in both primary and secondary health care settings using medication targets defined by Beers or STOPP criteria.

To replicate the intervention in different RCTs, a standard CDS tool could be developed. These CDS tools could promote communication between physicians and pharmaceutical servives. These RCTs could also assess adverse drug reactions, quality of life measurements, and patient and professional satisfaction, with a reasonable follow-up to clarify the clinical usefulness of these tools.

## Abbreviations

CDS: computerized decision support
NSAID: nonsteroidal anti-inflammatory drugs
PDDI: potential drug-drug interaction
PIM: potentially inappropriate medications
PIP: potentially inappropriate prescriptions
RCT: randomized controlled trial
